# *Subtercola endophyticus* sp. nov., a cold-adapted bacterium isolated from *Abies koreana*

**DOI:** 10.1038/s41598-022-16116-3

**Published:** 2022-07-15

**Authors:** Lingmin Jiang, Yuxin Peng, Jiyoon Seo, Doeun Jeon, Mi Gyeong Jo, Ju Huck Lee, Jae Cheol Jeong, Cha Young Kim, Hyeong Cheol Park, Jiyoung Lee

**Affiliations:** 1 Korean Collection for Type Cultures (KCTC), Biological Resource Center, Korea Research Institute of Bioscience and Biotechnology (KRIBB), Jeongeup, Jeollabuk-do 56212 Republic of Korea; 2 Team of Vulnerable Ecological Research, Division of Climate and Ecology, Bureau of Conservation & Assessment Research, National Institute of Ecology (NIE), Seocheon, 33657 Republic of Korea

**Keywords:** Microbiology, Plant sciences

## Abstract

A novel Gram-stain-positive, aerobic bacterial strain, designated AK-R2A1-2^ T^, was isolated from the surface-sterilized needle leaves of an *Abies koreana* tree. Strain AK-R2A1-2^ T^ had 97.3% and 96.7% 16S rRNA gene sequence similarities with *Subtercola boreus* K300^T^ and *Subtercola lobariae* 9583b^T^, respectively, but formed a distinct phyletic lineage from these two strains. Growth of strain AK-R2A1-2^ T^ was observed at 4–25 °C at pH 5.0–8.0. Strain AK-R2A1-2^ T^ contained menaquinone 9 (MK-9) and menaquinone 10 (MK-10) as the predominant respiratory quinones. The major cellular fatty acids were anteiso-C_15:0_ and summed feature 8 (C_18:1_ω7*c* or/and C_18:1_ω6*c*), and the polar lipids included diphosphatidylglycerol (DPG) and three unknown aminolipids, AKL2, AKL3, and AKL4. The complete genome of strain AK-R2A1-2^ T^ was sequenced to understand the genetic basis of its survival at low temperatures. Multiple copies of cold-associated genes involved in cold-active chaperon, stress response, and DNA repair supported survival of the strain at low temperatures. Strain AK-R2A1-2^ T^ was also able to significantly improve rice seedling growth under low temperatures. Thus, this strain represents a novel species of the genus *Subtercola*, and the proposed name is *Subtercola endophyticus* sp. nov. The type strain is AK-R2A1-2^ T^ (= KCTC 49721^ T^ = GDMCC 1.2921^ T^).

## Introduction

*Abies koreana* is a species of native fir that grows in the high mountains (1000–1900 m) of South Korea^1^. The tree is tolerant to alkaline soils and prefers to grow in cold locations with heavy winter snowfall^2^. *Abies koreana* was classified as endangered by the International Union for Conservation of Nature (IUCN) due to many unverified reasons leading to a massive decline in numbers and sudden dried death of these trees. This tree has potential applications in traditional medicine for the treatment of colds, stomachache, indigestion, and rheumatic disease^3^. Other important secondary metabolites of *Abies koreana*, such as essential oils, have antioxidant, antimicrobial, and anti-inflammatory properties^1,4^.

The genus *Subtercola*, a member of the family Microbacteriaceae within the order Micrococcales, was first characterized by Männistö et al.^5^ with the type species *Subtercola boreus* DSM 13056^ T^. In January 2022, this genus contained four child taxa with published names (https://lpsn.dsmz.de/genus/subtercola). Species of the genus *Subtercola* have been isolated from extremely high-altitude cold environmental niches, including groundwater^5^, snowy mountains^6^, and lakes^7^. Members of the genus grow optimally at low temperatures^5–7^ and are aerobic, Gram stain-positive, and non-endospore-producing, with pale to yellow, circular, convex, and smooth colonies. The polar lipids of this genus are phosphatidylglycerol, diphosphatidylglycerol, one unknown phospholipid, and two glycolipids^5^, the major fatty acid is anteiso-C_15:0_, and MK-9 and MK-10 are the major respiratory quinones^5–7^. The genus *Subtercola* has a high G + C content that ranges from 64.4 to 69.0% (https://ncbi.nlm.nih.gov/genome/?term=subtercola).

Omics approaches have facilitated the investigation of microbial diversity and plant host-endophyte interactions in multiple ecological communities. Many endophytes are able to synthesize novel compounds to maintain a mutualistic relationship with the host. A recent investigation of the bacterial community of *Abies koreana* trees sought to identify plant–microbe interactions and define important secondary metabolites. During this analysis, 81 endophytic strains were isolated from surface-sterilized needle leaves and one of the novel strains, designated AK-R2A1-2^ T^, was selected for further exploration in the present study. The taxonomic status of strain AK-R2A1-2^ T^ was evaluated using phenotypic, phylogenetic, genotypic, and chemotaxonomic data. Genome annotation indicated that this strain has multiple copies of cold-associated genes and the potential to produce secondary metabolites with antioxidant properties. The plant growth promotion effects of strain AK-R2A1-2^ T^ were assessed in rice as a cold-sensitive crop. The results showed that this novel *Subtercola* species isolated from *Abies koreana* in Mount Halla has potential value for crop cultivation.

## Results and discussion

### Phylogenetic analysis

**Figure 1:**
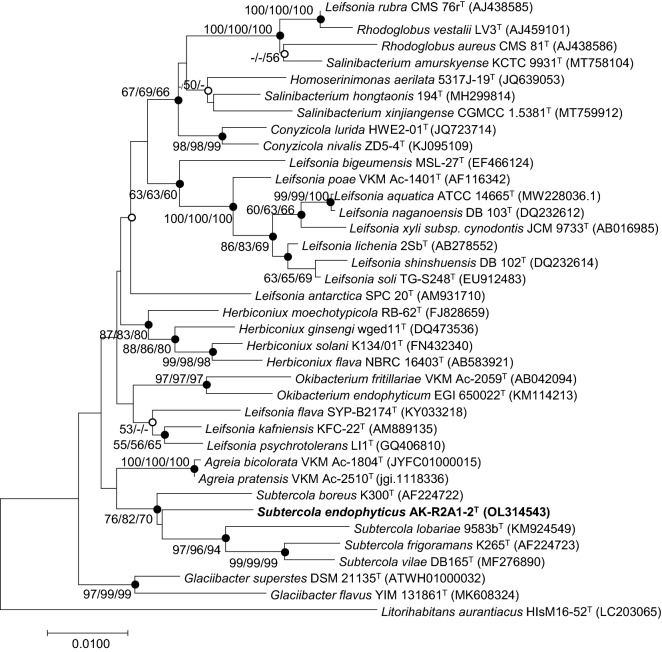
Neighbor-joining (NJ) phylogenetic tree based on 16S rRNA gene sequences showing the position of strain AK-R2A1-2^ T^. Bootstrap values (> 50%) were calculated using the NJ, maximum-likelihood (ML), and minimum evolution (ME) algorithms. Filled circles on the nodes indicate that the relationships were also identified using the ML and ME algorithms, whereas open circles indicate nodes were identified by either the ML or the ME algorithm. Scale bar: 0.0100 substitutions per nucleotide position.

The assembled 16S rRNA gene amplicon of strain AK-R2A1-2^ T^ had 1,457 base pairs (bp). Comparative sequence analysis showed that strain AK-R2A1-2^ T^ belonged to the genus *Subtercola* and, shared the highest 16S rRNA gene sequence similarity with *Subtercola boreus* DSM 13056^ T^ (97.3%), followed by 96.7% sequence similarity with *Agreia bicolorata* VKM Ac-1804^ T^, *Agreia pratensis* VKM Ac-2510^ T^, and *Subtercola lobariae* KCTC 33586^ T^ (all 96.7%). Sequence similarities with the other two members of the genus *Subtercola* were 96.2% and 96.1%. Strain AK-R2A1-2^ T^ was regarded as a novel species of the genus *Subtercola* based on its novel species recognition threshold value of 98.6%^8^. To identify the phylogenetic location of strain AK-R2A1-2^ T^, phylogenetic trees from 16S rRNA gene sequences were reconstructed using the MEGA program. The NJ phylogenetic tree showed that strain AK-R2A1-2^ T^ formed a cluster with *S. boreus* DSM 13056^ T^, and all *Subtercola* members comprised a single group. The ML and ME phylogenetic trees supported this result (Fig. 1). Thus, strain AK-R2A1-2^ T^ was confirmed as a member of the genus *Subtercola* rather than the genus *Agreia*. Based on the 16S rRNA gene sequence similarity and phylogenetic analysis, strains *S. boreus* DSM 13056^ T^, *S. vilae* DSM 105013^ T^, *Subtercola frigoramans* KCTC 49696^ T^, and *Subtercola lobariae* KCTC 33586^ T^ were selected for further comparative study under the same conditions.

### Phenotypic characteristics

Phenotypic characteristics based on the optimal growth medium, temperature, pH, and salt tolerance were investigated. Strain AK-R2A1-2^ T^ was shown to grow well on R2A, PDA, MEA, YEP, ISP2, and GYM medium, but did not on MA, LB, NA, and TSA. Cells were short and rod-shaped without flagella (0.2–0.3 µm in width and 0.3–1.6 µm in length, Fig. S1), and were Gram stain-positive, non-motile, catalase-positive, and oxidase-negative. Growth was observed in R2A medium at 4–25 °C (optimal 20 °C), pH 5–8 (optimal pH 5), and 0–1% NaCl (optimal 0%). Differential physiological characteristics that distinguish strain AK-R2A1-2^ T^ from closely related strains are shown in Table 1.

**Table 1:** Differential phenotypic characteristics that distinguish strain AK-R2A1-2^ T^ from closely related type strains. Strains: 1, AK-R2A1-2^ T^; 2, *Subtercola lobariae* KCTC 33586^ T^; 3, *Subtercola frigoramans* KCTC 49696^ T^; 4, *Subtercola boreus* DSM 13056^ T^; 5, *Subtercola vilae* DSM 105013^ T^. All data are from the present study unless indicated otherwise. + , Positive; w, weakly positive; − , negative; ND, not detected.

Characteristic	1	2	3	4	5
Isolation source	*Abies koreana*	Lichen^a^	Boreal groundwater^b^	Boreal groundwater^b^	Cold volcano lake^c^
Colony color	White, circular	White, circular^a^	Pale yellow to bright yellow^b^	Yellow, circular^b^	Golden yellow^c^
Cell size (µm)	(0.2–0.3) × (0.3–1.6)	(0.5–0.6) × (0.7–1.0) ^a^	(0.3–0.4) × (0.9–1.5)^b^	(0.2–0.3) × (0.6 − 1.0)^b^	0.5 × (1.0–1.2)^c^
**Growth conditions**					
Temperature range (optimum)(°C)	4–25 (20)	4–28 (20)^a^	2–28 (15–17)^b^	2–28 (15–17) ^b^	5–28 (10–15)^c^
NaCl tolerance (optimum) (%, w/v)	0–1 (0)	ND	ND	ND	0^c^
pH range (optimum)	5.0–8.0 (5)	4.0–8.0 (6)^a^	ND	ND	5.0–8.0 (7)^c^
Major menaquinone	MK-9, MK-10	MK-10^a^	MK-9, MK-10^b^	MK-9, MK-10^b^	MK-9, MK-10^c^
G + C content (mol%)	65.8	66.8^a^	67.8^b^	64^c^	65^d^
**API 20NE**					
Indole production	−	−	−	+	−
Esculin hydrolysis	+	+	+	w	+
β-Galactosidase	+	−	+	−	−
Glucose assimilation	−	−	+	−	−
**API ZYM**					
Alkaline phosphate	−	−	+	−	w
Esterase(C4)	+	+	+	+	w
Lipase (C14)	w	−	−	w	−
Valine arylamidase	w	w	w	−	w
Cystine arylamidase	w	w	w	−	−
Trypsin	w	−	w	−	−
β-Galactosidase	w	−	+	+	−
α-Mannosidase	w	+	−	−	−
**API 50CH**					
Glycerol	−	−	−	−	w
D-Arabinose	−	−	−	−	w
L-Arabinose	−	−	−	−	w
D-Xylose	−	−	w	−	w
L-Xylose	−	−	w	w	w
D-Adonitol	−	−	w	w	w
Methyl-βD-xylopyranoside	−	−	w	w	w
D-Galactose	−	−		+	w
D-Glucose	−	−	w	−	w
D-Fructose	−	−	−	−	w
L-Sorbose	−	−	−	−	w
L-Rhamnose	−	−	−	−	w
Inositol	−	−	−	−	w
D-Mannitol	−	−	−	−	w
*N*-Acetylglucosamine	−	−	−	w	−
Amygdalin	−	−	w	w	−
Salicin	−	−	w	w	w
D-Cellobiose	−	−	w	w	w
Maltose	−	−	w	w	w
D-Lactose	−	−	+	w	w
D-Melezitose	−	−	w	+	+
D-Saccharose	−	−	w	−	−
D-Trehalose	−	−	w	−	−
Gentiobiose	−	−	−	−	w
Potassium 2-keto-gluconate	−	−	−	−	w

^a^Si et al., 2017, ^b^Männistö et al., 2000, ^c^Villalobos et al., 2017.

### Chemotaxonomic features

The cellular fatty acid profiles (> 1%) of strain AK-R2A1-2^ T^ and closely related strains are shown in Table 2. The major fatty acids (> 10%) of strain AK-R2A1-2^ T^ were anteiso-C_15:0_ (12.3%) and summed feature 8 (76.7%). This was most similar to *S. lobariae* KCTC 33586^ T^, which also contained anteiso-C_15:0_ (25.8%) and summed feature 8 (52.1%) as its major fatty acids. The major fatty acids of *S. boreus* DSM 13056^ T^ were anteiso-C_15:0_ (46.0%), iso-C_15:0_ (20.7%), 2-OH C_14:0_ (10.4%), and summed feature 8 (11.8%), while those of *S. vilae* DSM 105013^ T^ were anteiso-C_15:0_ (64.1%), iso-C_16:0_ (12.6%), and 2-OH C_14:0_ (15.6%), and those of *S. frigoramans* KCTC 49696^ T^ were summed feature 8 (40.4%) and summed feature 3 (30.2%). Differences in major and minor fatty acids can differentiate strain AK-R2A1-2^ T^ from other closely related members of the genus *Subtercola* (Table 2). The respiratory quinones of strain AK-R2A1-2^ T^ were menaquinone 9 (MK-9) and menaquinone 10 (MK-10), which is similar to other closely related *Subtercola* members. The major polar lipids in strain AK-R2A1-2^ T^ were diphosphatidylglycerol (DPG) and three unknown aminolipids, AKL2, AKL3, and AKL4. The polar lipid profile was more simple for strain AK-R2A1-2^ T^ than for strains of other *Subtercola* species (Fig. S2).

**Table 2:** Cellular fatty acid profiles (> 1%) of strain AK-R2A1-2^ T^ and type strains of closely related species. Strains: 1, AK-R2A1-2^ T^; 2, *Subtercola lobariae* KCTC 33586^ T^; 3, *Subtercola frigoramans* KCTC 49696^ T^; 4, *Subtercola boreus* DSM 13056^ T^; 5, *Subtercola vilae* DSM 105013^ T^. Values are percentages of total fatty acids. ND, not detected. Major components (> 10%) are shown in bold. All data were obtained from the present study.

	1	2	3	4	5
C_14:0_	ND	ND	2.7	0.2	ND
C_16:0_	ND	ND	8.5	6.9	2.0
C_18:0_	ND	ND	ND	ND	0.5
iso-C_15:0_	ND	ND	ND	**20.7**	ND
iso-C_16:0_	3.9	8.0	ND	ND	**12.6**
anteiso-C_15:0_	**12.3**	**25.8**	1.6	**46.0**	**64.1**
anteiso-C_17:0_	1.9	3.6	ND	ND	5.1
2OH-C_14:0_	ND	3.9	9.9	**10.4**	**15.6**
C_17:1_ ω9c	5.2	4.9	ND	ND	1.3
C_16:1_ ω5c	ND	ND	2.1	ND	ND
C_18:1_ ω7c 11-methyl	ND	ND	2.2	0.2	ND
Summed feature*					
3	ND	ND	**30.2**	ND	ND
8	**76.7**	**52.1**	**40.4**	**11.8**	0.8

*Summed features are groups of two or three fatty acids that cannot be separated by GLC using the MIDI System. Summed feature 3 contains C_16:1_*ω*6*c* and/or C_16:1_*ω*7*c*; summed feature 8 is listed as C_18:1_*ω*7*c* and/or C_18:1_*ω*6*c*.

### Genome analyses

#### *General and functional features of the AK-R2A1-2*^* T*^* genome*

**Figure 2:**
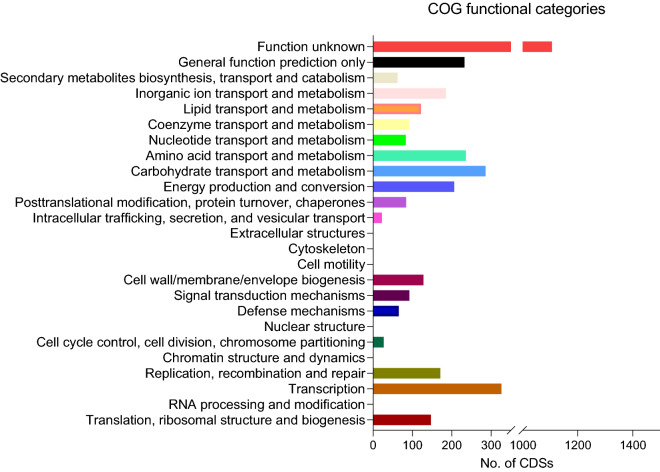
The cluster of orthologous groups (COG) classification of putative proteins in the whole genome sequence of strain AK-R2A1-2^ T^.

After assembly using the Canu (version 1.7) de novo assembler, the whole genome of strain AK-R2A1-2^ T^ was shown to comprise a single circular chromosome of 4,318,731 bp, and the N50 value was 12,176 bases, with coverage of 95x. The G + C content calculated based on the respective whole-genome sequence was 65.8%. A comparison between two copies of the 16S rRNA gene fragments with the whole-genome sequence showed that DNA sequence contamination did not occur during the genome assembly of strain AK-R2A1-2^ T^. The whole genome sequence of the novel strain was deposited into NCBI under accession number CP087997.1. The genome sequence was annotated using RAST^9,10^, and protein-coding sequences were determined using PGAP^11^. The NCBI PGAP annotation revealed that the AK-R2A1-2^ T^ genome contained 3,874 protein-coding genes and 56 RNA genes, including two 5S rRNA, two 16S rRNA, two 23S rRNA, three non-coding RNA (ncRNA), and 47 tRNA genes (Table S1). A total of 3,674 protein-coding sequences (CDSs) were annotated using the cluster orthologous group (Fig. 2). The largest group of CDSs was classified as unknown (1,106 of the total CDSs, 30.1%), while the groups with the lowest CDSs numbers were classified as cytoskeleton and RNA processing and modification (1 of the total CDSs, 0.02%). Transcription (8.9%) and carbohydrate transport and metabolism (7.8%) gene had the next highest numbers of CDSs annotated from the whole genome (Fig. 2 and S3).

**Figure 3:**
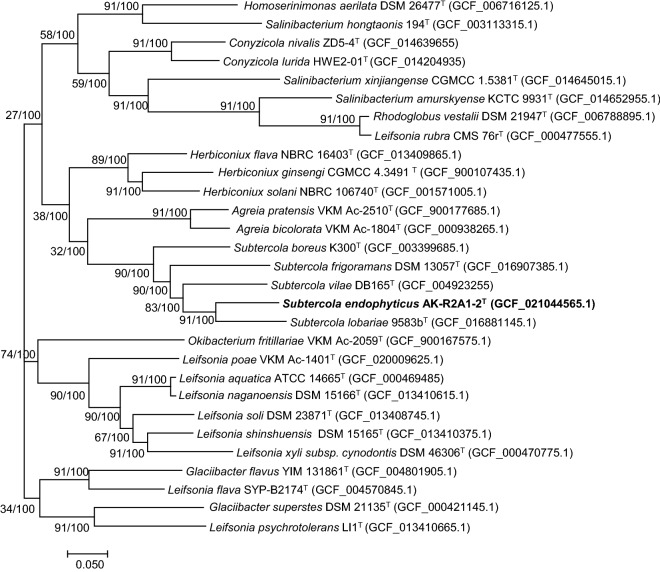
ML phylogenomic tree based on UBCGs (concatenated alignment of 92 core genes). Gene support index (GSI, left) and bootstrap values (right) are indicated at the nodes. Scale bar, 0.050 substitutions per position.

A whole genome-based phylogenetic tree was reconstructed using UBCG (version 3.0) and showed that strain AK-R2A1-2^ T^ forms a subgroup with *S. lobariae* 9583b^T^, and that all members of the genus *Subtercola* comprise one group (Fig. 3). Concurrently, members of the genus *Agreia* form a single cluster, consistent with the phylogenetic tree created using 16S rRNA gene sequences. These findings suggested that strain AK-R2A1-2^ T^ was a novel species of the genus *Subtercola*.

#### *Genomic features associated with the cold-adaption of strain AK-R2A1-2*^* T*^

The RAST server, PGAP, and BlastKOALA pipeline were used to perform genome annotation and determine the metabolic pathways of strain AK-R2A1-2^ T^. The following information is predicted from the genome sequence analyses:

*Stress response* Members of the genus *Subtercola* grow well at low temperatures, and while strain AK-R2A1-2^ T^ was isolated at 25 °C, it was shown to grow at 4 °C; the type strains of other *Subtercola* members can even grow at − 2°C^5^. The potential mechanism(s) by which bacteria invoke stress responses to adapt to environmental changes have not yet been fully elucidated. Bacterial adaptations to facilitate survival at low temperatures include cell membrane fluidity (increased unsaturated fatty acids) and protein-specific responses (refolding cold-damaged proteins)^12^. Cold-shock proteins (Csp) are produced by bacteria in response to a rapid decrease in temperature and act as RNA chaperones to help prevent mRNA misfolding^13^. The PGAP analysis in the current study showed that the AK-R2A1-2^ T^ genome contained two genes encoding a cold-shock protein (UFS59601.1; UFS60376.1) and a cold shock domain-containing protein (UFS59609.1). When whole-genome mining of strain AK-R2A1-2^ T^ was performed using RAST SEED and BlastKOALA, many well-studied, cold-inducible genes^14–20^ were annotated by the BlastKOALA pipeline, including *aceE* (encoding pyruvate dehydrogenase E1 component [EC:1.2.4.1]), *aceF* (pyruvate dehydrogenase E2 component, dihydrolipoamide acetyltransferase [EC:2.3.1.12]), *cspA* (cold shock protein), *deaD* (ATP-dependent RNA helicase DeaD [EC:3.6.4.13]), *dnaA* (chromosomal replication initiator protein), *gyrA* (DNA gyrase subunit A [EC:5.6.2.2]), *hupB* (DNA-binding protein HU-beta), *infA* (translation initiation factor IF-1), *infB* (translation initiation factor IF-2), *infC* (translation initiation factor IF-3), *nusA* (transcription termination/antitermination protein NusA), *otsA* (trehalose 6-phosphate synthase [EC:2.4.1.15 2.4.1.347]), *otsB* (trehalose 6-phosphate phosphatase [EC:3.1.3.12]), *pnp* (polyribonucleotide nucleotidyltransferase [EC:2.7.7.8]), *rbfA* (ribosome-binding factor A), *recA* (recombination protein RecA), and *tig* (trigger factor) (Table 3). Compatible solutes, a group of compounds that are important for osmotic stress and cold shock, can reduce membrane stress using various mechanisms. Biosynthetic genes of the recognized cold-protective solutes, glycine betaine (*opuC, opuBD, opuA*), choline (*betaA*), and proline (*proA, proB, proC*) were annotated^21,22^. In addition, genes responsible for the synthesis of the osmoprotectant proline from glutamate (*proA*, glutamate-5-semialdehyde dehydrogenase [EC:1.2.1.41]; *proB*, glutamate 5-kinase [EC:2.7.2.11]; *proC* (pyrroline-5-carboxylate reductase; [EC:1.5.1.2]), two choline dehydrogenases (*betA*, [EC:1.1.99.1]), and the glycine betaine transport system (*opuC*, *opuBD*, *opuA*) were identified in the AK-R2A1-2^ T^ genome^23–27^ (Table 3). Using the RAST SEED annotation of strain AK-R2A1-2^ T^ (Fig. S4), 22 genes were associated with the stress response. Of these, three were involved in osmotic stress (osmoregulation), 11 were related to oxidative stress [oxidative stress (6), glutathione: redox cycle (3), glutaredoxins (1), glutathionylspermidine and trypanothione (1)], and eight were related to detoxification. Some freezing-protective solutes, which can contribute to cold stress remission^28,29^, such as amino sugar and nucleotide sugars (26), amino acids (96), starch, and sucrose (27) were also detected in the genome information from strain AK-R2A1-2^ T^.

**Table 3:** Genes encoding known cold-stress response molecules as predicted in the AK-R2A1-2^ T^ genome.

Gene name^a^	Description
*aceE*	Pyruvate dehydrogenase E1 component [EC:1.2.4.1]
*aceF*	Pyruvate dehydrogenase E2 component [EC:2.3.1.12]
*cspA*	Cold shock protein
*deaD*	ATP-dependent RNA helicase DeaD [EC:3.6.4.13])
*dnaA*	Chromosomal replication initiator protein
*gyrA*	DNA gyrase subunit A [EC:5.6.2.2]
*hupB*	DNA-binding protein HU-beta
*infA*	Translation initiation factor IF-1
*infB*	Translation initiation factor IF-2
*infC*	Translation initiation factor IF-3
*nusA*	Transcription termination/anti-termination protein NusA
*otsA*	Trehalose 6-phosphate synthase [EC:2.4.1.15 2.4.1.347]
*otsB*	Trehalose 6-phosphate phosphatase [EC:3.1.3.12]
*pnp*	Polyribonucleotide nucleotidyltransferase [EC:2.7.7.8]
*rbfA*	Ribosome-binding factor A
*recA*	Recombination protein RecA
*tig*	Trigger factor
*proA*	Glutamate-5-semialdehyde dehydrogenase [EC:1.2.1.41]
*proB*	Glutamate 5-kinase [EC:2.7.2.11]
*proC*	Pyrroline-5-carboxylate reductase; [EC:1.5.1.2]
*betA*	Choline dehydrogenase (betA, [EC:1.1.99.1]
*opuC*^*b*^	Glycine betaine transport system
*opuA*^*b*^	Glycine betaine transport system
*opuBD*^*b*^	Glycine betaine transport system

^a^Barria, et al., 2013; ^b^Hoffmann, et al., 2011.

*Motility* Bacterial motility is critical to its establishment in and colonization of its host, and promotes plant growth^30^. Two genes related to motility, one for flagellar assembly (*rpoD*) and one for bacterial chemotaxis (*rbsB*), were annotated from the whole genome of strain AK-R2A1-2^ T^. The incomplete motility pathway of strain AK-R2A1-2^ T^ explains why it was non-motile.

*Fatty acid metabolism* Membrane fatty acids are the major determinants for a sufficiently fluid membrane state required for low-temperature membrane function. The melting point of membrane fatty acids at cold temperatures is reduced by the synthesis of short-chain fatty acids, increases in the relative abundance of mono/polyunsaturated fatty acids, and increases in the anteiso/iso branched-chain fatty acids ratio^31–34^. The beta-oxidation-related degradation pathway, including 3-hydroxyacyl-CoA dehydrogenase [EC:1.1.1.35], acyl-CoA oxidase [EC:1.3.3.6], acyl-CoA dehydrogenase [EC:1.3.8.7], acetyl-CoA acyltransferase [EC:2.3.1.16], and enoyl-CoA hydratase [EC:4.2.1.17]^35^, was detected in the whole genome of strain AK-R2A1-2^ T^. In addition, fatty acid biosynthesis genes, *fabD*, *fabF*, *fabG*, *fabH*, *fabL*, *OXSM*, *OAR1*, and unsaturated fatty acid biosynthesis-related genes including *tesB* were detected in the whole genome.

### Genome comparison and biosynthetic potential of the novel strain

The proposed threshold values of dDDH and ANI for delineating novel bacterial species are < 70% and < 95–96%, respectively^36^. In the current study, the ANI values were 76.8–80% between strain AK-R2A1-2^ T^ and other members of the genus *Subtercola,* 73.6–74% between AK-R2A1-2^ T^ and members of the genus *Agreia*, and < 74.7% between AK-R2A1-2^ T^ and other genera in the family Microbacteriaceae. Consistently, strain AK-R2A1-2^ T^ exhibited a dDDH value of 22.5–24.5% with members of the genus *Subtercola*, 20.1–20.5% with members of the genus *Agreia*, and < 20.7% with other genera in the family Microbacteriaceae (Table S2). Similar results were obtained for orthoANI values, which were < 80.3% with most closely related strains in the family Microbacteriaceae (Table S2).

Bacteria produce a large number of secondary metabolites that act as a reservoir of bioactive metabolites, with some exhibiting unique functions such as salt resistance^37^. In the genus *Subtercola*, there are limited studies on the production of specialized metabolites and the potential source of natural products remains untapped. In recent years, many computational methods have been developed to identify biosynthetic gene clusters (BGCs) in genomic data. AntiSMASH is a platform that is widely used for genome mining of secondary metabolites. The secondary metabolite clusters of the AK-R2A1-2^ T^ genome were searched using antiSMASH (V. 6.0.1). Seven clusters were identified from the whole genome and found to contain non-alpha poly-amino acids like ε-polylysine, microansamycin (polyketide), alkylresorcinol (polyketide), kosinostatin (NPR + polyketide), beta-lactone, carotenoid (terpene), and lipopolysaccharide (saccharide: lipopolysaccharide) (Table S3 and Figs. S5–S11). Some of these compounds have vital antioxidant, antimicrobial, and anti-inflammatory properties. For example, NAPAA is a cationic peptide shown to prevent microbe proliferation and is approved as a food-grade cationic antimicrobial metabolite^38^, which has natural antioxidant and antimicrobial activities^39^. Carotenoid can be used to protect against oxidative stress in model systems^40–43^, and regulates membrane fluidity at low temperature using cell membrane adaptation^44,45^. Higher carotenoid levels may also increase resistance to cold shock^46^. Alkylresorcinol can protect epithelial cells against oxidative damage and has antioxidant^47–49^, antigenotoxic^49^ and potential antiglioma activity^50^. Kosinostatin has antitumor, antimicrobial, and antiproliferative functions^51,52^, while beta-lactone is a natural product with significant clinical applications provided by its antimicrobial, anticancer, and anti-obesity properties^53,54^. Microansamycin is a member of the macrolactams family, important bioactive compounds with anti-tuberculous and anti-tumor activity^55^. Similarly, its host, the Korean fir, produces rich polyphenol compounds like quercetin (flavonoid) and carotenoids to provide the strong antioxidant properties of Korean fir^56^. The essential oil of Korean fir includes the terpene group (monoterpenes, oxygenated monoterpenes, sesquiterpenes, diterpenes, and limonene)^57,58^ which also have strong antioxidant, antimicrobial, and anti-inflammatory activities^1,4^. The functional secondary metabolites of strain AK-R2A1-2^ T^ were very similar to its host plant.

### Effect of strain AK-R2A1-2^ T^ on rice seedling growth under low temperature

**Figure 4:**
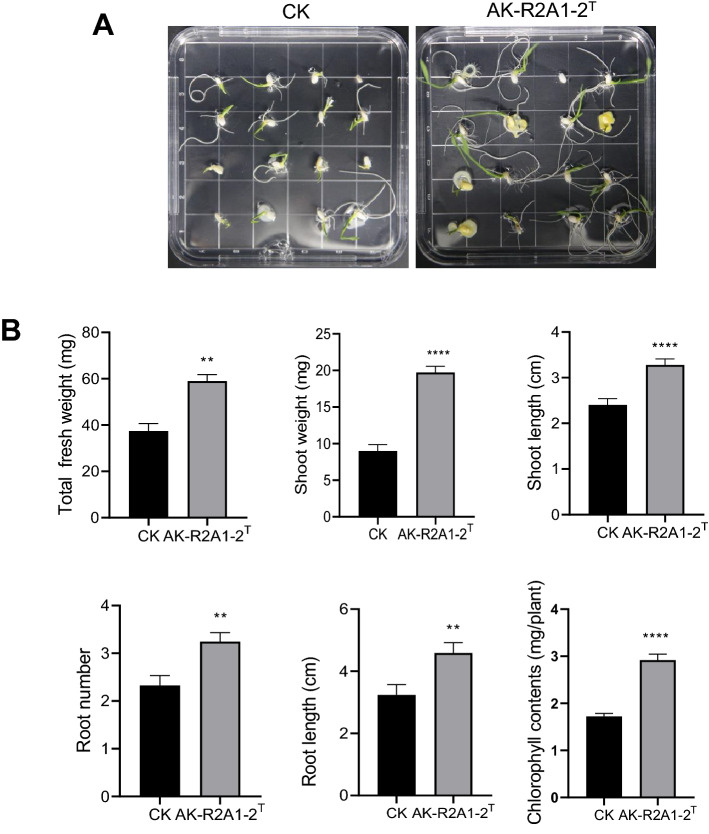
Effects of coating strain AK-R2A1-2^ T^ on rice growth under low temperature. (**A**) Comparison of rice seedling growth in response to a control (CK, R2A broth) or AK-R2A1-2^ T^ bacterial suspension (1 × 10^8^ cfu/mL) on water agar plates at 20 °C for 10 days. (**B**) Quantification of total fresh weight, shoot weight, shoot length, root number, root length, and chlorophyll contents. Error bars indicate the standard deviation of the mean (*n* = 15). Asterisks indicate a statistically significant difference between the control and coating bacterial suspension (two-way ANOVA, **p < 0.01, and ****p < 0.0001). Experiments were repeated twice with similar results.

Many endophytic bacteria have a broad host range. For example, *Bacillus altitudinis,* isolated from the wild plant species, *Glyceria chinensis*, can promote the growth of *Arabidopsis thaliana*, *N. tabacum*, corn, and soybean^59^. *Bacillus megaterium* RmBm31, an endophytic bacterium isolated from *Retama monosperma*, increases the biomass of *A. thaliana*^60^. Rice is a cold-sensitive crop, and its exposure to low-temperature stress (< 20 °C) during germination and early seedling growth, can have a negative impact on initial stand establishment^61,62^. To assess the impact of AK-R2A1-2^ T^ on rice seedling growth under low temperatures, rice seeds were co-cultivated with strain AK-R2A1-2^ T^ for 10 days at 20 °C, and total fresh weight, shoot weight, shoot length, root number, root length, and chlorophyll contents were examined in the rice seedling (Fig. 4). Shoot weight and shoot length were 1.67-fold and 1.36-fold higher, respectively, in the AK-R2A1-2^ T^-exposed seedlings than in controls (CK), while root length and root number were 1.42-fold and 1.40-fold higher, respectively, in the AK-R2A1-2^ T^-exposed seedings (Fig. 4). Strain AK-R2A1-2^ T^ significantly improved rice seedling biomass and root morphological parameters under low temperatures. Whole genome analysis showed that these improvements were associated with phytohormone biosynthesis genes for indole-3-acetic acid (*trpABCDES*) and phosphate solubilization (*pstABCS*, *phoHLU*, and *phnB*). Indole-3-acetic acid can induce the growth of auxin-dependent lateral root formation, root hair development, and primary root growth, which promote plant growth. Meanwhile, phosphate-solubilizing bacteria enhance plant production by solubilizing insoluble phosphorus to make it available and increasing phosphorus nutrition. Secondary metabolites including terpenes were also shown to promote plant growth and some BGCs may produce new secondary metabolites because they display low similarity to known clusters. The function of these secondary metabolites on plant growth will require further study.

## Conclusion

According to phylogenetic, phenotypic, and chemotaxonomic data, strain AK-R2A1-2^ T^ was shown to belong to the genus *Subtercola*. The differentiating phenotypic properties and chemotaxonomic data presented in Table 1 preliminarily distinguish this strain as a novel species. Genome analyses of strain AK-R2A1-2^ T^ using RAST SEED, BlastKOALA, and antiSMASH identified genes for a cold-active chaperone, osmotic, and oxidative stress responses, and DNA repair mechanisms, as well as the gene clusters responsible for host plant secondary metabolites. These data support the physiological properties of the psychrophilic genus *Subtercola*, demonstrate bacterial and plant viability at low temperatures, and define the secondary metabolites isolated from the host plant.

### Description of *Subtercola endophyticus* sp. nov

*Subtercola endophyticus* (en.do.phy’ti.cus. Gr. pref. *endo-*, within; Gr. neut. n. *phyton*, plant; L. masc. adj. suff. *-icus*, adjectival suffix used with the sense of belonging to; N.L. masc. adj. *endophyticus*, endophytic, within plants, pertaining to the original isolation from plant tissues).

Colonies grown on R2A medium are white, circular (1–4 mm in diameter), smooth, and convex after a 4 day culture at 25 °C. Cells (0.2–0.3 µm in width and 0.3–1.6 µm in length) are short rod-shaped, strictly aerobic, Gram stain-positive, non-motile, lacking flagella, catalase-positive, and oxidase-negative. Growth occurs at 4–25 °C (optimum, 20 °C), at pH 5–8 (optimal 5), and with 0–1% NaCl (optimal 0%). Strain AK-R2A1-2^ T^ grows on R2A, PD, MEA, YEP, ISP2, and GYM (optimum, PD, and R2A), but not on MA, LB, NA, and TSA medium. Strain AK-R2A1-2^ T^ was positive for the following enzyme activities in API ZYM strips: esterase (C4), esterase lipase (C8), lipase (C14), leucine arylamidase, valine arylamidase, cysteine arylamidase, trypsin, acid phosphate, naphthol-AS-BI-phosphohydrolase, β-galactosidase, α-glucosidase, β-glucosidase, and α-mannosidase. In the API 20NE strips, strain AK-R2A1-2^ T^ was positive for aesculin hydrolysis and β-galactosidase, but negative for all other activities. In the API 50CH strips, all tests were negative except aesculin ferric citrate. The major fatty acids detected in strain AK-R2A1-2^ T^ were summed featured 8 and anteiso-C_15:0_, the respiratory quinones were MK-9 and MK-10 and the major polar lipids were DPG, AKL2, AKL3, and AKL4.

The GenBank accession numbers of the 16S rRNA gene and whole genome sequences of strain AK-R2A1-2^ T^ are OL314543 and CP087997.1, respectively. The strain is available from the Korea Collection for Type Culture (KCTC 49721^ T^) and the Guangdong Microbial Culture Collection Center (GDMCC 1.2921^ T^).

## Materials and methods

### Sample isolation and culture conditions

Plant samples were collected from the top region of Mount Halla, Jeju Island, Republic of Korea (33°21′32.4″ N, 126°31′68″ E) in July 2020. Two grams of needle leaves from *Abies koreana* tree samples were surface-sterilized with 1% sodium hypochlorite, rinsed five times in sterile distilled water, and crushed with 20 mL of 1 × phosphate-buffered saline (PBS) in a grinder. Ground samples were serially diluted from 10^−1^ to 10^−4^ with 1 × PBS, and 100 µL aliquots were spread onto tenfold diluted Reasoner’s 2A agar (R2A, Difco), and incubated at 25 °C for 7 days. Morphologically distinct colonies were selected and subsequently streaked on fresh R2A medium. All strains were preserved in sterile skimmed milk (10%, w/v) at − 80 °C. Among all the isolates, one white, circular, convex, and smooth colony, designated AK-R2A1-2^ T^, was selected for further study. *Subtercola boreus* DSM 13056^ T^(=K300^T^), *Subtercola vilae* DSM 105013^ T^(=DB165^T^), *Subtercola frigoramans* KCTC 49696^ T^(=K265^T^), and *Subtercola lobariae* KCTC 33586^ T^ (=9583b^T^) were obtained from the corresponding culture collections as closely related strains to analyze the taxonomic characteristics under comparable culture conditions. Unless otherwise stated, bacterial strains were grown on R2A or ISP2 for 5 days for the subsequent tests.

### Strain identification and phylogenetic analysis

The 16S rRNA gene of strain AK-R2A1-2^ T^ was amplified by PCR using universal primers 27F (5′-AGAGTTTGATCMTGGCTCAG-3′) and 1492R (5′-TACGGYTACCTTGTTACGACTT-3′) with bacterial DNA as the template. The purified PCR product was sequenced by BioFact (Daejeon, Republic of Korea) using primers 27F, 1492R, 518F (5′-CCAGCAGCCGCGGTAATACG-3′), and 800R (5′-TACCAGGGTATCTAATCC-3′). Sequencing reads were assembled using Vector NTI software (1.6.1), and an almost full-length 16S rRNA gene sequence was obtained and subjected to similarity-based BLAST analyses against the GenBank (https://blast.ncbi.nlm.nih.gov/Blast.cgi) and EzBioCloud (http://www.ezbiocloud.net)^36^ databases. Phylogenetic trees based on 16S rRNA gene sequences were reconstructed with the Molecular Evolutionary Genetics Analysis (MEGA V.7.0) program^63^, using the neighbor-joining (NJ), minimum evolution (ME), and maximum-likelihood (ML) algorithms with 1,000 bootstrap iterations. *Litorihabitans aurantiacus* HIsM16-52^ T^ (GenBank accession number LC203065) was included as an outgroup.

### Phenotypic characteristics

AK-R2A1-2^ T^ cell morphology was observed by scanning electron microscopy at the Chuncheon Center, Korea Basic Science Institute (KBSI) after growing the strain on R2A agar plates for 4 days. Gram staining was conducted with a Gram staining kit (Difco) according to the manufacturer’s instructions. Cell motility was tested by growing the strain on a semi-solid R2A medium (0.4%). Catalase activity was determined by the production of bubbles after adding 3% (v/v) hydrogen peroxide solution to fresh cells^64^, while oxidase activity was measured using oxidase test strips (bioMérieux) that changed color to purple. Different media including R2A, potato dextrose agar (PDA, Difco), malt extract agar (MEA, Difco), yeast extract peptone agar (YEP, Difco), ISP medium No.2 (ISP2, Difco), and GYM medium (GYM: glucose 4 g/L, yeast extract 4.0 g/L, malt extract 10 g/L, CaCO_3_ 2 g/L, agar 15 g/L), marine agar 2216 (MA, Difco), Luria–Bertani agar (LB, Difco), nutrient agar (NA, Difco), and trypticase soy agar (TSA, Difco) were screened for optimal growth of the tested strains. Various temperatures (4, 10, 15, 20, 25, 30, 37, 40, 45, and 60 °C) were assayed to identify the growth range of the novel strain. The pH range (3.0 to 12.0, adjusted with 1 N HCl and NaOH) for growth and NaCl tolerance (0 to 15%, w/v; 1% concentration increments) were measured in R2A liquid medium, monitoring the optical density at 600 nm (OD_600_) using a microplate spectrophotometer (Multiskan skyhigh, Thermo Fisher Scientific). Other biochemical features, such as the utilization of substrates, acid production from carbohydrates, and enzyme activities, were tested using API 20NE (bioMérieux) or API ZYM with NaCl 0.85% medium (bioMérieux) and API 50CH according to the manufacturer’s protocols. All closely related type strains were tested under the same conditions.

### Chemotaxonomy features

Biomass for analysis of cellular fatty acids from strain AK-R2A1-2^ T^ and closely related type strains was extracted from cells grown in R2A media. Using the Sherlock Microbial Identification System version 6.0 (MIDI) standard protocol, whole-cell fatty acid methyl esters were extracted and analyzed by gas chromatography (model 6890 N; Agilent) using the Microbial Identification software package^65^. Quinones were extracted from 100 mg of freeze-dried cells by shaking in chloroform: methanol (2:1, v/v) and purified by thin-layer chromatography as described by Collins et al.^66^. Analysis of the purified quinones was performed using reverse-phase high-performance liquid chromatography (HPLC) with ultraviolet (UV) absorbance detection at 270 nm. For extraction and analysis of polar lipids, 100 mg of freeze-dried cells were boiled in methanol, mixed and shaken with chloroform, evaporated, and identified by two-dimensional thin-layer chromatography (TLC) on Kieselgel 60 F254 plates (silica gel, 10 × 10 cm; Merck). Lipids spots were visualized by spraying with 0.2% ninhydrin (Sigma-Aldrich), α-naphthol, molybdenum blue (Sigma-Aldrich), 4% phosphomolybdic acid reagent, and Dragendorff’s solution to detect amino group-containing, sugar-containing, phosphorus-containing, total, and quaternary nitrogen-containing lipids, respectively.

### Genome sequencing and assembly

Whole genome sequencing was performed by the Macrogen facility (Macrogen, Republic of Korea) using the PacBio Sequel System (Pacific Biosciences, Inc.) and the Illumina sequencing platform. Raw sequencing data were assembled using the Canu (version 1.7) de novo assembler^67^. Error corrections were performed by Pilon (version 1.21)^68^. Potential contamination of the assembled genome was checked using the ContEst16S algorithm^69^ to compare 16S rRNA within the whole genome. To verify that strain AK-R2A1-2^ T^ belonged to the genus *Subtercola*, a whole genome-based phylogenetic tree was reconstructed using the up-to-date bacterial core gene set and pipeline (UBCG) as described by Na et al.^70^.

### Genome annotation and comparative analysis

The National Center for Biotechnology Information Prokaryotic Genome Annotation Pipeline (PGAP) and Rapid Annotation of microbial genomes using Subsystem Technology (RAST) SEED^9–11^ were used for genome annotation. Metabolic pathways were reconstructed using BlastKOALA, which is based on the Kyoto Encyclopedia of Genes and Genomes (KEGG) pathway database^71^. Genome mining for the presence of secondary metabolite gene clusters was performed using the antiSMASH program (https://antismash.secondarymetabolites.org/) with a “relaxed detection strictness” parameter and a “known cluster blast” feature including non-ribosomal peptide synthetases (NRPSs), type I and type II polyketide synthases (PKSs), lanthipeptides, lasso peptides, sactipeptides, and thiopeptides^72^. Comparative genome analyses including digital DNA-DNA hybridization (dDDH), average nucleotide identity (ANI) values, and orthoANI values between strain AK-R2A1-2^ T^ and the most closely related members of the family Microbacteriaceae were performed using the Genome-to-Genome Distance Calculation (GGDC) webserver (http://ggdc.dsmz.de/)^73^, ANI calculator (https://www.ezbiocloud.net/), and standalone Orthologous Average Nucleotide Identity (OAT) software^74^, respectively. A graphical circular map of the AK-R2A1-2^ T^ genome was constructed using CGView (https://cgview.ca/).

### Rice growth and low-temperature conditions

Seeds of the *Japonica* rice cultivar Haepung were surface-sterilized with 1% (v/v) sodium hypochlorite for 10 min followed by repetitive washes with distilled water and soaked in AK-R2A1-2^ T^ suspension (1 × 10^8^ CFU/mL) or R2A broth as a control as described as Jiang et al.^37^. The control and AK-R2A1-2^ T^ bacteria-coated seeds were incubated in the dark at 14 °C for 4 days in three replicated square plates (245 mm × 245 mm) containing 0.15% water agar. All the plates were incubated at 20 °C with a 16 h light/8 h dark photoperiod for 10 days. To estimate shoot weight, shoot length, root weight, root number, and root length, fifteen seedling plants from each experiment were considered. Chlorophyll contents were calculated as described by Fu et al.^75^. All experiments were performed in triplicate yielding similar results.

## Supplementary Information

Supplementary Information.

## Data Availability

The strain is available from the Korean Collection for Type Cultures (KCTC 49721^T^) and the Guangdong Microbial Culture Collection Center (GDMCC 1.2921^T^). The GenBank accession number of AK-R2A1-2^T^ for the 16S rRNA gene is OL314543. The whole genome sequence of AK-R2A1-2^T^ accession number is CP087997.1. The associated BioSample and BioProject accession numbers are SAMN23075623 and PRJNA678113, respectively. The taxonomy ID is 2,895,559.
