# Supplementary material for: Subtercola endophyticus sp. nov., a cold-adapted bacterium isolated from Abies koreana
Source: Sci Rep. 2022 Jul 15;12:12114. doi: 10.1038/s41598-022-16116-3 (PMC9287328; doi:10.1038/s41598-022-16116-3)
Supplement: Supplementary file 1 — Supplementary Information. [file 41598_2022_16116_MOESM1_ESM.pdf]

Supplementary Information

Subtercola endophyticus sp. nov., a cold-adapted bacterium isolated from *Abies koreana*

Lingmin Jiang[1], Yuxin Peng[1], Jiyoon Seo[1], Doeun Jeon[1], Mi Gyeong Jo[1], Ju Huck Lee[1], Jae Cheol Jeong[1], Cha Young Kim[1], Hyeong Cheol Park[2], Jiyoung Lee[1,*]

[1]Korean Collection for Type Cultures (KCTC), Biological Resource Center, Korea Research Institute of Bioscience & Biotechnology (KRIBB), Jeongeup, Jeollabuk-do 56212, Republic of Korea

[2]Team of Vulnerable Ecological Research, Division of Climate and Ecology, Bureau of Conservation & Assessment Research, National Institute of Ecology (NIE), Seocheon, 33657, Republic of Korea

[*] Corresponding author: J.Lee; jiyoung1@kribb.re.kr

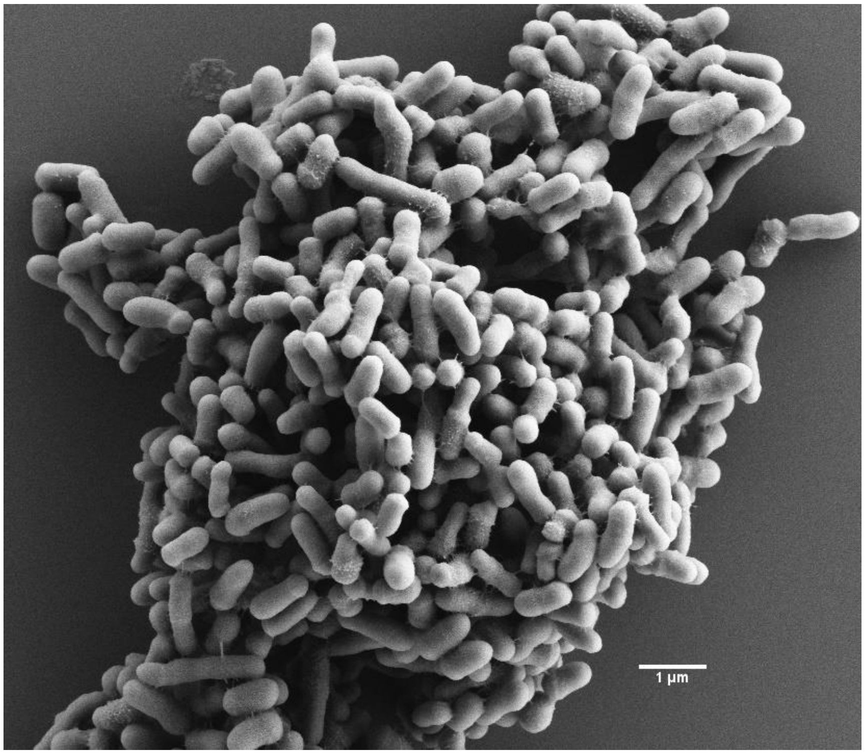

Fig. S1. Scanning electron micrograph of strain AK-R2A1-2^T. Scale bar, 1 µm.

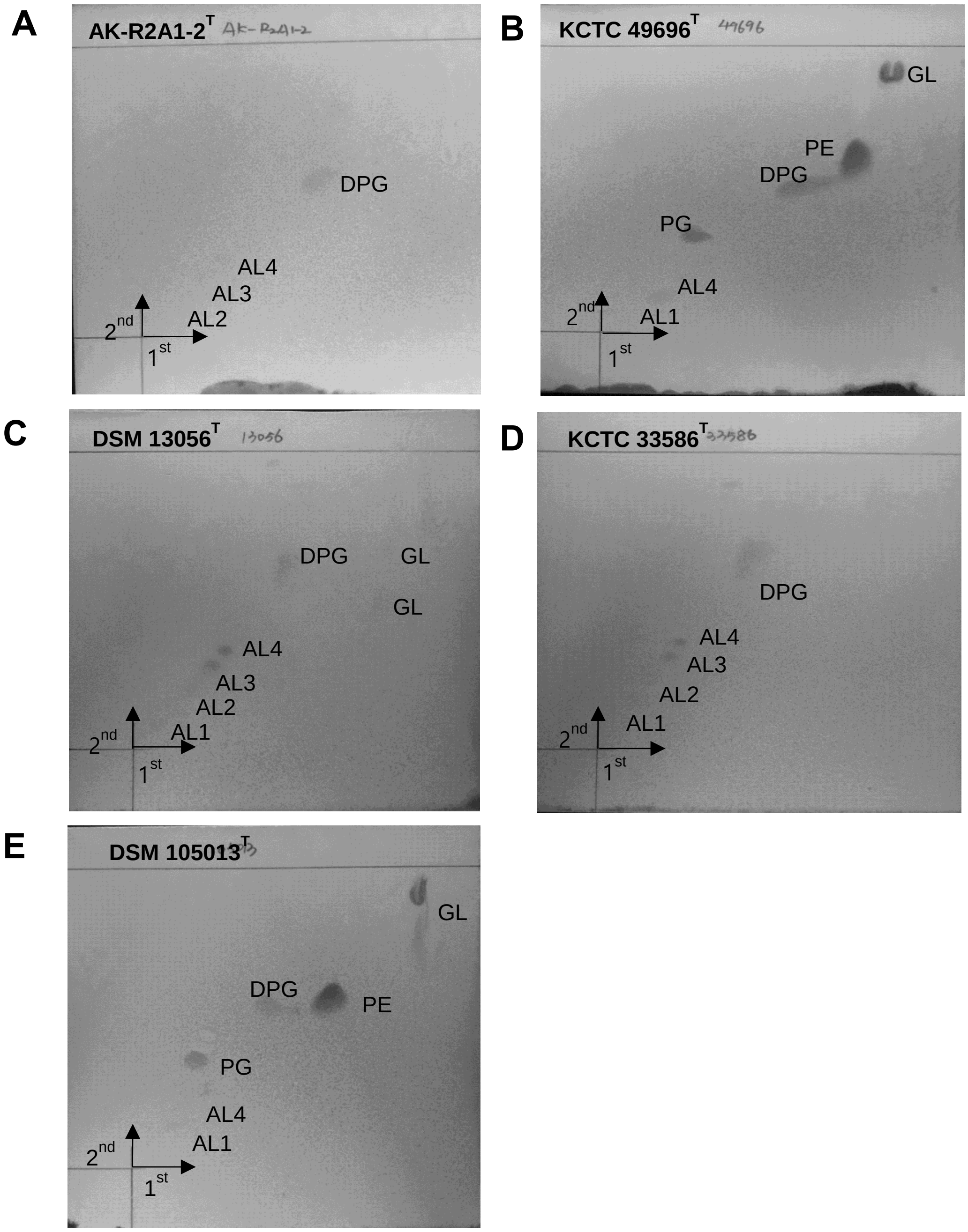

Fig. S2. Polar lipid profile of strain AK-R2A1-2^{T} and closely related type strains.

A) AK-R2A1-2^{T}; B) *Subtercola frigoramans* KCTC 49696^{T}; C) *Subtercola boreus* DSM 13056^{T}; D) *Subtercola lobariae* KCTC 33586^{T}; E) *Subtercola vilae* DSM 105013^{T}. DPG, diphosphatidylglycerol, PE, phosphatidylethanolamine; AL, unknown aminolipid; GL, unknown glycolipid.

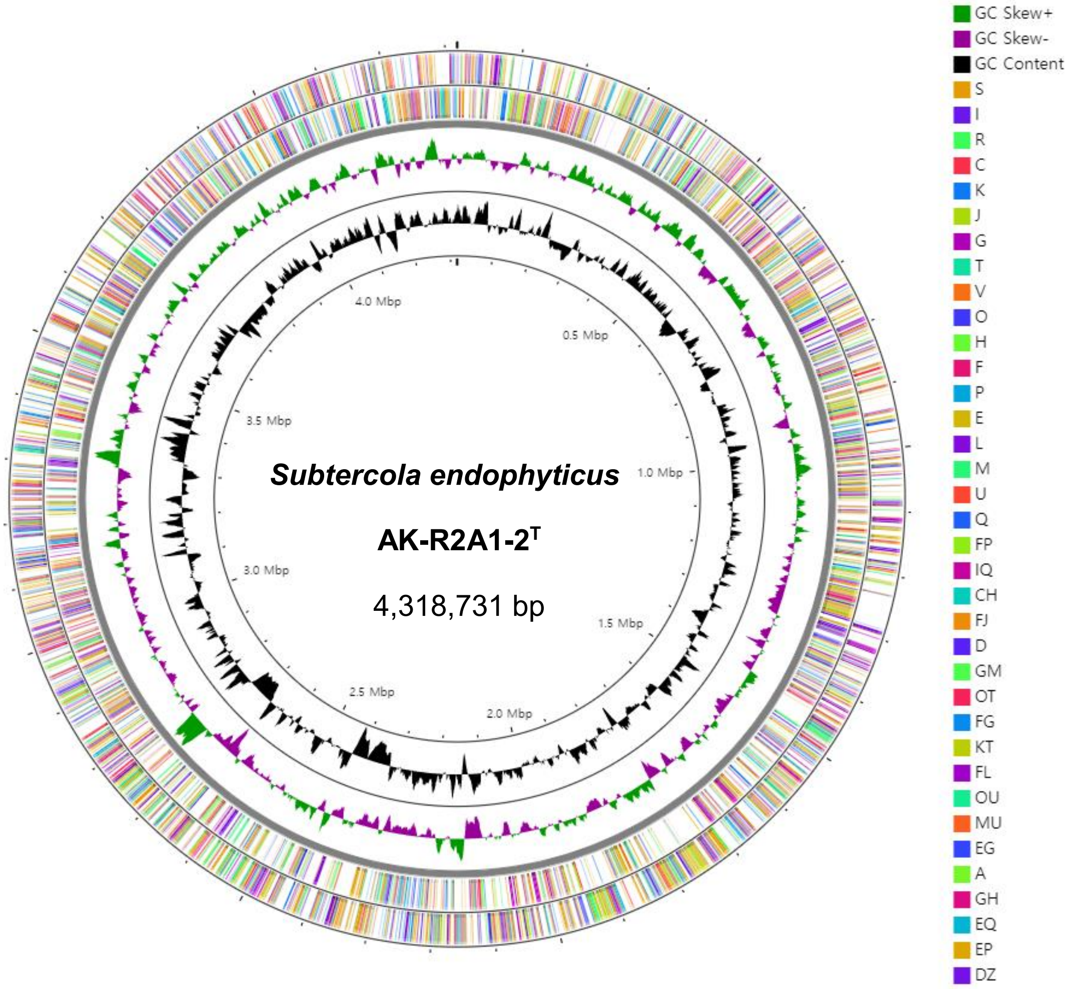

Fig. S3. Map of the AK-R2A1-2^{T} genome generated with CGView. Marked characteristics are shown from the outside to the center. Rings 1 and 2 show cluster orthologous group (COG) annotation in the forward and reverse directions, respectively. Ring 3 shows the GC skew, while ring 4 shows the G+C % content plot. The COG categories are: A, RNA processing and modification; B, chromatin structure and dynamics; C, energy production and conversion; D, cell cycle control, cell division, and chromosome partitioning; E, amino acid transport and metabolism; F, nucleotide transport and metabolism; G, carbohydrate transport and metabolism; H, coenzyme transport and metabolism; I, lipid transport and metabolism; J, translation, ribosomal structure, and biogenesis; K, transcription; L, replication, recombination, and repair; M, cell wall/membrane/envelope biogenesis; N, cell motility; O, post-translational modification, protein turnover, chaperones; P, inorganic ion transport and metabolism; Q, secondary metabolite biosynthesis, transport, and catabolism; R, general function prediction only; S, function unknown; T, signal transduction mechanisms; U, intracellular trafficking, secretion, and vesicular transport; V, defense mechanisms; W, extracellular structures; X, mobilome: prophages, transposons; and Z, cytoskeleton.

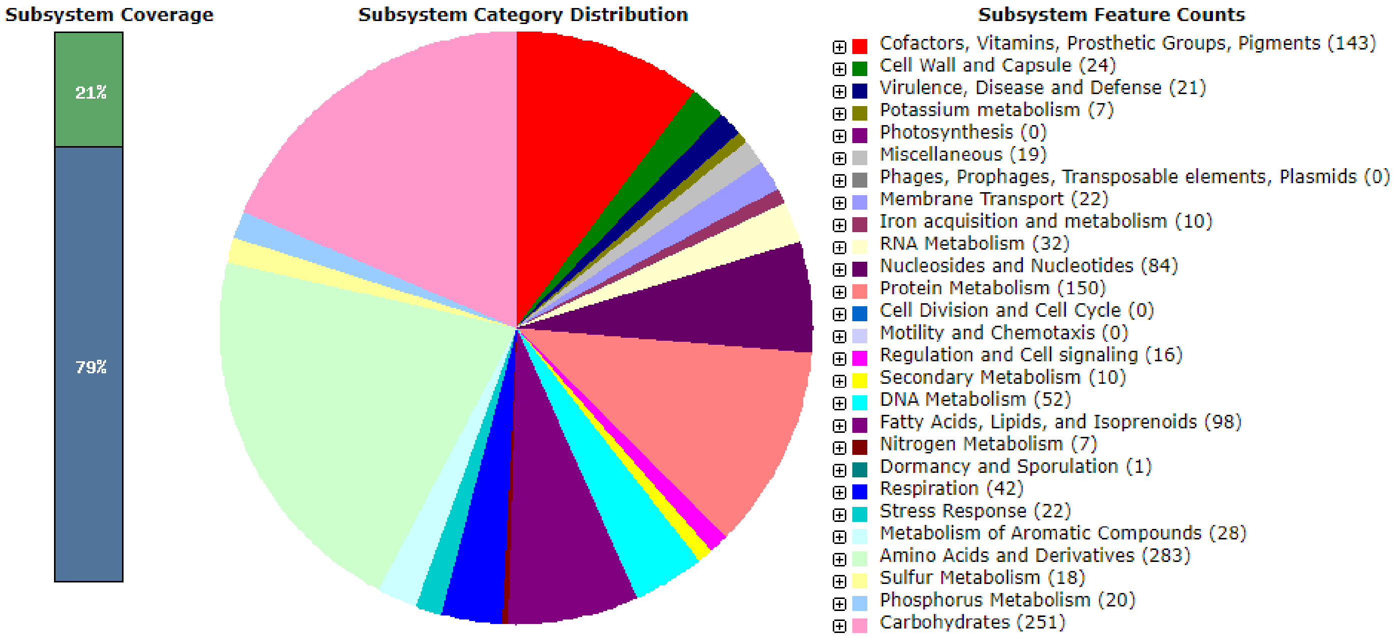

Fig. S4. Subsystem features of strain AK-R2A1-2^{T} revealed by the RAST server.

A

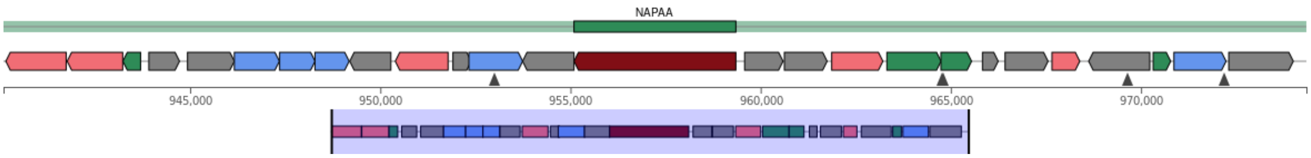

B

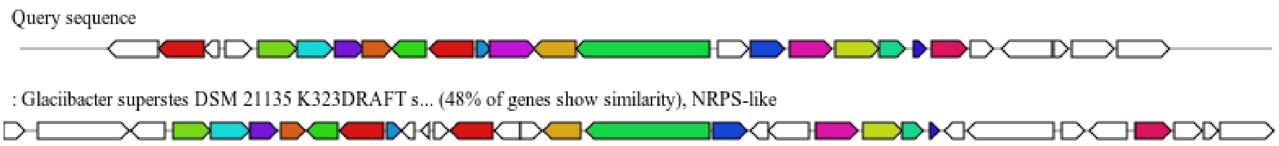

C

Contig No./locus tag	Identified protein
ctg1_922	FAD dependent oxidoreductase
ctg1_923	Aminotransferase class-III
ctg1_924	AsnC-type helix-turn-helix domain
ctg1_925	Protein phosphatase 2C
ctg1_926	Bacterial extracellular solute-binding protein
ctg1_927	ABC transporter
ctg1_928	Binding-protein-dependent transport system inner membrane component
ctg1_929	Binding-protein-dependent transport system inner membrane component
ctg1_930	L-asparaginase II
ctg1_931	Aminotransferase class-III
ctg1_932	OsmC-like protein
ctg1_933	Major Facilitator Superfamily
ctg1_934	Peptidase M1 N-terminal domain
ctg1_935	AMP-binding enzyme
ctg1_936	FKBP-type peptidyl-prolyl cis-trans isomerase
ctg1_937	1-deoxy-D-xylulose 5-phosphate reductoisomerase
ctg1_938	Peptidase family M50
ctg1_939	Histidine kinase
ctg1_940	Response regulator receiver domain
ctg1_941	No significant similarity
ctg1_942	GcpE protein
ctg1_943	Acetyltransferase (GNAT) domain
ctg1_944	Domain of unknown function (DUF222)
ctg1_945	MarR family
ctg1_946	Transmembrane secretion effector
ctg1_947	Trypsin-like peptidase domain

Fig. S5. Information of non-alpha poly-amino acids like ε-polylysine (NAPAA) biosynthetic gene cluster of strain AK-R2A1-2^T predicted by antiSMASH. A) Identified secondary metabolite regions using strictness “relaxed” option for detection. B) Type I polyketide synthase (TIPKS) metabolite clusterblast compared with the NCBI database. C) Pfam-based GO term annotation of genes. Maroon highlighting denotes core biosynthetic genes, while pink highlighting shows additional biosynthetic genes.

A

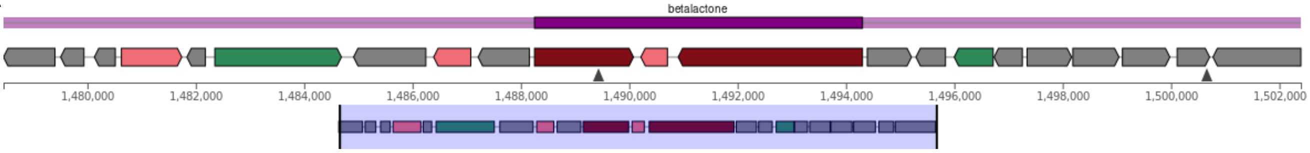

B

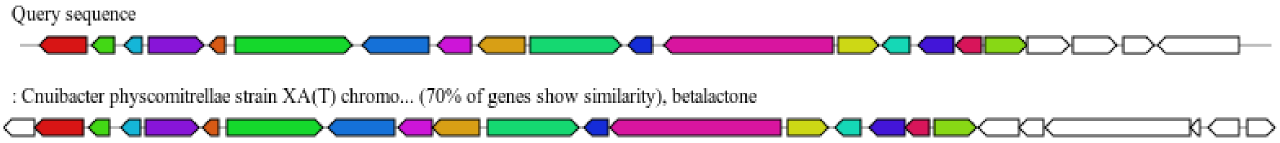

C

Contig No./locus tag	Identified protein
ctg1_1392	MraW methylase family
ctg1_1393	MraZ protein, putative antitoxin-like
ctg1_1394	Protein of unknown function (DUF3040)
ctg1_1395	Polyprenyl synthetase
ctg1_1396	Rv2175c C-terminal domain of unknown function
ctg1_1397	Protein kinase domain
ctg1_1398	Class-II DAHP synthetase family
ctg1_1399	Acyltransferase
ctg1_1400	ROK family
ctg1_1401	AMP-binding enzyme
ctg1_1402	Polypeptide deformylase
ctg1_1403	Biotin carboxylase, N-terminal domain
ctg1_1404	AAA domain
ctg1_1405	MerR HTH family regulatory protein
ctg1_1406	MerR HTH family regulatory protein
ctg1_1407	FHA domain
ctg1_1408	Protein of unknown function (DUF1295)
ctg1_1409	Protein of unknown function (DUF3097)
ctg1_1410	Amino-transferase class IV
ctg1_1411	UPF0126 domain
ctg1_1412	Trypsin-like peptidase domain

Fig. S6. Information of microansamycin biosynthetic gene cluster of strain AK-R2A1-2^T predicted by antiSMASH. A) Identified secondary metabolite regions using strictness “relaxed” option for detection. B) Type I polyketide synthase (TIPKS) metabolite clusterblast compared with the NCBI database. C) Pfam-based GO term annotation of genes. Maroon highlighting denotes core biosynthetic genes, while pink highlighting shows additional biosynthetic genes.

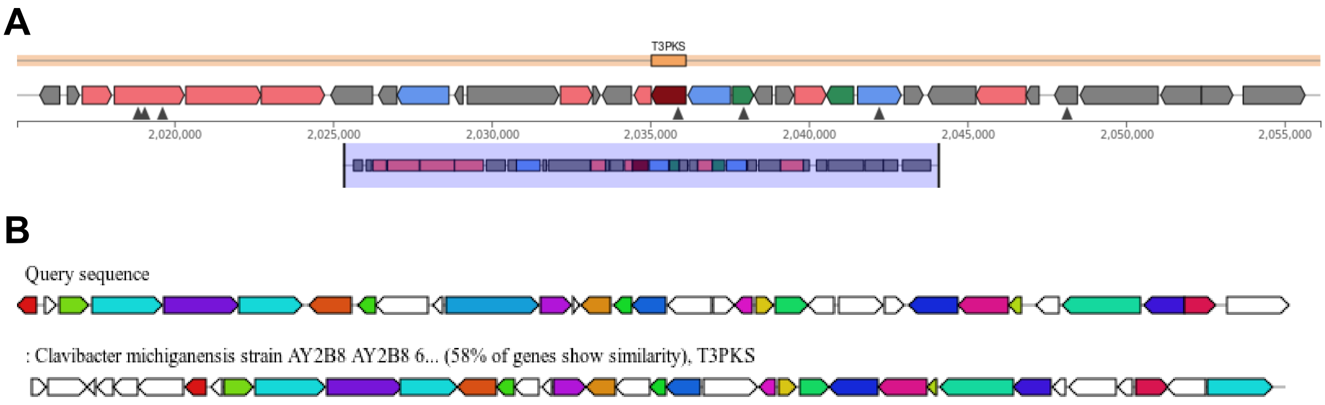

C

Contig No./locus tag	Identified protein
ctg1_1888	ABC-type cobalt transport system, permease component
ctg1_1889	No significant similarity
ctg1_1890	Luciferase-like monooxygenase
ctg1_1891	Carbohydrate-binding module 48 (Isoamylase N-terminal domain)
ctg1_1892	Alpha amylase, catalytic domain
ctg1_1893	Alpha amylase, catalytic domain
ctg1_1894	ImpB/mucB/samB family
ctg1_1895	Protein of unknown function (DUF1697)
ctg1_1896	ABC transporter
ctg1_1897	No significant similarity
ctg1_1898	SNARE associated Golgi protein
ctg1_1899	Aldo/keto reductase family
ctg1_1900	No significant similarity
ctg1_1901	Uncharacterized protein conserved in bacteria (DUF2236)
ctg1_1902	Isoprenylcysteine carboxyl methyltransferase (ICMT) family)
ctg1_1903	Chalcone and stilbene synthases, N-terminal domain)
ctg1_1904	Major Facilitator Superfamily
ctg1_1905	Bacterial regulatory proteins, gntR family
ctg1_1906	Acetyltransferase (GNAT) family
ctg1_1907	Mycothiol maleylpyruvate isomerase N-terminal domain
ctg1_1908	short chain dehydrogenase
ctg1_1909	Bacterial regulatory helix-turn-helix protein, lysR family
ctg1_1910	Transmembrane secretion effector
ctg1_1911	Protein of unknown function (DUF1345)
ctg1_1912	GatB/GatE catalytic domain
ctg1_1913	Amidase
ctg1_1914	Glu-tRNAGln amidotransferase C subunit
ctg1_1915	No significant similarity
ctg1_1916	NAD-dependent DNA ligase adenylation domain
ctg1_1917	tRNA methyl transferase
ctg1_1918	Endonuclease/Exonuclease/phosphatase family
ctg1_1919	Bacterial protein of unknown function (DUF853)

Fig. S7. Information of alkylresorcinol biosynthetic gene cluster of AK-R2A1-2^T predicted by antiSMASH. A) Identified secondary metabolite regions using strictness “relaxed” option for detection. B) Type I polyketide synthase (TIPKS) metabolite clusterblast compared with the NCBI database. C) Pfam-based GO term annotation of genes. Maroon highlighting denotes core biosynthetic genes, while pink highlighting shows additional biosynthetic genes.

A

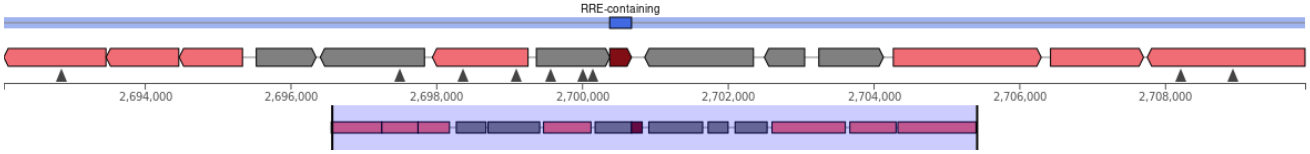

B

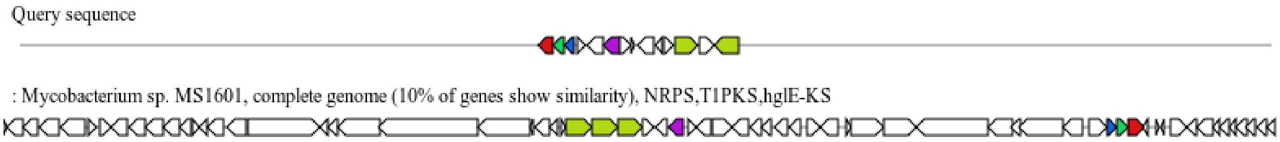

C

Contig No./locus tag	Identified protein
ctg1_2495	dTDP-4-dehydrorhamnose 3,5-epimerase
ctg1_2496	NAD dependent epimerase/dehydratase family
ctg1_2497	Nucleotidyl transferase
ctg1_2498	No significant similarity
ctg1_2499	Malate:quinone oxidoreductase (Mqo)
ctg1_2500	UDP-glucose/GDP-mannose dehydrogenase family, NAD binding domain
ctg1_2501	Uncharacterized nucleotidyltransferase
ctg1_2502	Coenzyme PQQ synthesis protein D (PqqD)
ctg1_2503	FAD dependent oxidoreductase
ctg1_2504	DUF218 domain
ctg1_2505	No significant similarity
ctg1_2506	Acyltransferase family
ctg1_2507	Polysaccharide biosynthesis protein
ctg1_2508	Acyltransferase family

Fig. S8. Information of kosinostatin biosynthetic gene cluster of AK-R2A1-2^T predicted by antiSMASH. A) Identified secondary metabolite regions using strictness “relaxed” option for detection. B) Type I polyketide synthase (TIPKS) metabolite clusterblast compared with the NCBI database. C) Pfam-based GO term annotation of genes. Maroon highlighting denotes core biosynthetic genes, while pink highlighting shows additional biosynthetic genes.

A

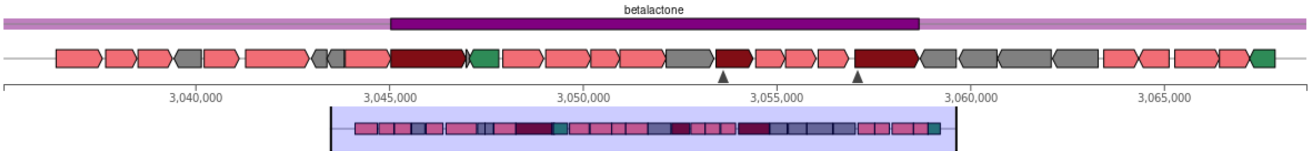

B

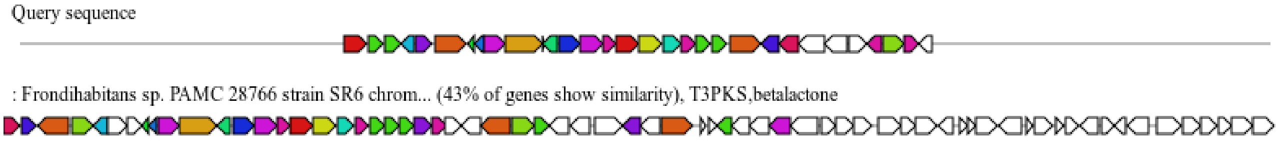

C

Contig No./locus tag	Identified protein
ctg1_2804	Thiolase, N-terminal domain
ctg1_2805	Enoyl-CoA hydratase/isomerase
ctg1_2806	Enoyl-CoA hydratase/isomerase
ctg1_2807	Thioesterase superfamily
ctg1_2808	Enoyl-(Acyl carrier protein) reductase
ctg1_2809	AMP-binding enzyme
ctg1_2810	MaoC like domain
ctg1_2811	N-terminal half of MaoC dehydratase
ctg1_2812	Acyl-CoA dehydrogenase, N-terminal domain
ctg1_2813	Acetyl-coenzyme A synthetase N-terminus
ctg1_2814	No significant similarity
ctg1_2815	Bacterial regulatory proteins, gntR family
ctg1_2816	Acyl-CoA dehydrogenase, N-terminal domain
ctg1_2817	Acyl-CoA dehydrogenase, N-terminal domain
ctg1_2818	Enoyl-(Acyl carrier protein) reductase
ctg1_2819	Thiolase, N-terminal domain
ctg1_2820	Acetyl-CoA hydrolase/transferase N-terminal domain
ctg1_2821	HMGL-like
ctg1_2822	Enoyl-(Acyl carrier protein) reductase
ctg1_2823	Enoyl-CoA hydratase/isomerase
ctg1_2824	Enoyl-CoA hydratase/isomerase
ctg1_2825	AMP-binding enzyme
ctg1_2826	PaaX-like protein
ctg1_2827	BadF/BadG/BcrA/BcrD ATPase family
ctg1_2828	2-hydroxyglutaryl-CoA dehydratase, D-component
ctg1_2829	2-hydroxyglutaryl-CoA dehydratase, D-component
ctg1_2830	Alpha/beta hydrolase family
ctg1_2831	Enoyl-(Acyl carrier protein) reductase
ctg1_2832	Acyl-CoA dehydrogenase, N-terminal domain
ctg1_2833	short chain dehydrogenase
ctg1_2834	Bacterial regulatory proteins, tetR family

Fig. S9. Information of beta-lactone biosynthetic gene cluster of AK-R2A1-2^T predicted by antiSMASH. A) Identified secondary metabolite regions using strictness “relaxed” option for detection. B) Type I polyketide synthase (TIPKS) metabolite clusterblast compared with the NCBI database. C) Pfam-based GO term annotation of genes. Maroon highlighting denotes core biosynthetic genes, while pink highlighting shows additional biosynthetic genes.

A

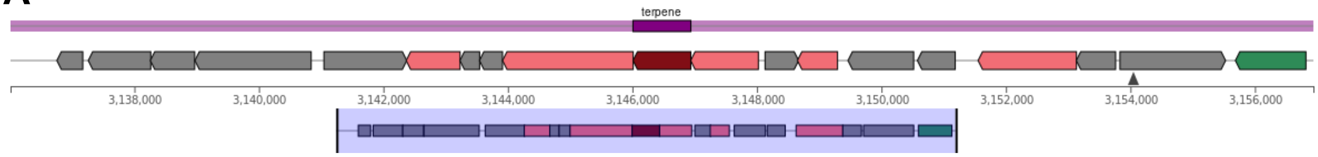

B

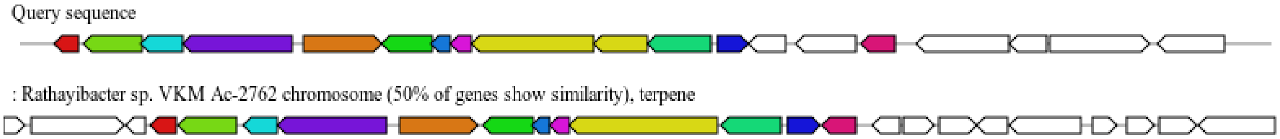

C

Contig No./locus tag	Identified protein
ctg1_2905	MerR HTH family regulatory protein
ctg1_2906	DnaJ domain
ctg1_2907	GrpE
ctg1_2908	Hsp70 protein
ctg1_2909	Vacuole effluxer Atg22 like
ctg1_2910	UbiA prenyltransferase family
ctg1_2911	CarR_dom_SF: lycopene cyclase domain
ctg1_2912	CarR_dom_SF: lycopene cyclase domain
ctg1_2913	Flavin containing amine oxidoreductase
ctg1_2914	Squalene/phytoene synthase
ctg1_2915	Polyprenyl synthetase
ctg1_2916	NUDIX domain
ctg1_2917	Haloacid dehalogenase-like hydrolase
ctg1_2918	No significant similarity
ctg1_2919	dUTPase
ctg1_2920	FAD-binding domain
ctg1_2921	No significant similarity
ctg1_2922	PAP2 superfamily
ctg1_2923	Bacterial regulatory helix-turn-helix protein, lysR family

Fig. S10. Information of carotenoid biosynthetic gene cluster of AK-R2A1-2^{T} predicted by antiSMASH. A) Identified secondary metabolite regions using strictness "relaxed" option for detection. B) Type I polyketide synthase (TIPKS) metabolite clusterblast compared with the NCBI database. C) Pfam-based GO term annotation of genes. Maroon highlighting denotes core biosynthetic genes, while pink highlighting shows additional biosynthetic genes.

A

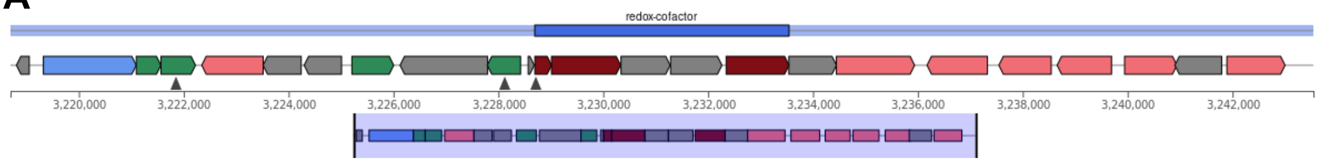

B

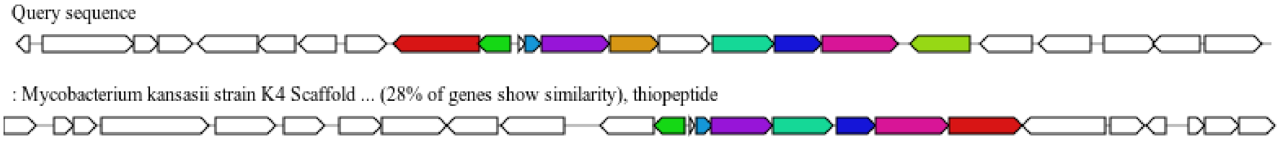

C

Contig No./locus tag	Identified protein
ctg1_2981	RelE-like toxin of type II toxin-antitoxin system HigB
ctg1_2982	Major Facilitator Superfamily
ctg1_2983	MarR family
ctg1_2984	Bacterial regulatory proteins, tetR family
ctg1_2985	Thiolase, N-terminal domain
ctg1_2986	Coenzyme A transferase
ctg1_2987	Coenzyme A transferase
ctg1_2988	IclR helix-turn-helix domain
ctg1_2989	GMC oxidoreductase
ctg1_2990	Bacterial regulatory proteins, tetR family
ctg1_2991	Mycofactocin: mycofactocin precursor)
ctg1_2992	Mycofact_MftB: putative mycofactocin binding protein MftB
ctg1_2993	Radical SAM superfamily
ctg1_2994	AAA domain (dynein-related subfamily)
ctg1_2995	Von Willebrand factor type A domain
ctg1_2996	FMN-dependent dehydrogenase
ctg1_2997	Creatinine amidohydrolase
ctg1_2998	Glycosyl transferase family 2
ctg1_2999	NADH:flavin oxidoreductase / NADH oxidase family)
ctg1_3000	Luciferase-like monooxygenase
ctg1_3001	Alcohol dehydrogenase GroES-like domain
ctg1_3002	Alcohol dehydrogenase GroES-like domain
ctg1_3003	HpcH/HpaI aldolase/citrate lyase family
ctg1_3004	NAD dependent epimerase/dehydratase family

Fig. S11. Information of lipopolysaccharide biosynthetic gene cluster of AK-R2A1-2^T predicted by antiSMASH. A) Identified secondary metabolite regions using strictness "relaxed" option for detection. B) Type I polyketide synthase (TIPKS) metabolite clusterblast compared with the NCBI database. C) Pfam-based GO term annotation of genes. Maroon highlighting denotes core biosynthetic genes, while pink highlighting shows additional biosynthetic genes.

Table S1. General features of *Subtercola endophyticus* AK-R2A1-2^{T}.

Feature	*Subtercola endophyticus* AK-R2A1-2^{T}
GenBank sequence accession ID	CP087997.1
**Genome assembly**	
Assembly method	Canu; V1.7
Sequencing technology	PacBio Sequel, Illumina platform
Annotation	NCBI PGAP
**Genome features**	
Genome length (bp)	4,318,731
G+C content (mol%)	65.8
No. of contigs	1
Total no. of genes	3,945
Protein-coding genes	3,874
Pseudogenes	71
RNA genes	56
rRNA genes (5S, 16S, 23S)	6 (2, 2, 2)
tRNA genes	47
ncRNA genes	3

Table S2. ANI (%), orthoANI (%), and dDDH (%) values between strain AK-R2A1-2^{T} and closely related type strains of members of the family *Microbacteriaceae*. Data were calculated from ANI calculator at EzBioCloud, Orthologous Average Nucleotide Identity (OAT) software, and GGDC web server, respectively.

Assembly No.	Strain	ANI	orthoANI	dDDH
GCF_016881145	*Subtercola lobariae* 9583b^{T}	80.0	80.3	24.5
GCF_004923255	*Subtercola vilae* DB165^{T}	77.6	78.2	22.6
GCF_003399685	*Subtercola boreus* K300^{T}	77.5	77.8	22.5
GCF_016907385	*Subtercola frigoramans* DSM 13057^{T}	76.8	77.5	22.5
GCF_013409865	*Herbiconiux flava* DSM 26474^{T}	74.7	74.0	20.7
GCF_001571005	*Herbiconiux solani* NBRC 106740^{T}	74.5	74.1	20.5
GCF_900107435	*Herbiconiux ginsengi* CGMCC 4.3491^{T}	74.4	73.6	20.6
GCF_000938265	*Agreia bicolorata* VKM Ac-1804^{T}	74.0	73.5	20.5
GCF_000469485	*Leifsonia aquatica* ATCC 14665^{T}	73.9	72.9	20.3
GCF_900177685	*Agreia pratensis* VKM Ac-2510^{T}	73.6	73.5	20.1
GCF_013410375	*Leifsonia shinshuensis* DSM 15165^{T}	73.4	72.5	19.9
GCF_013410615	*Leifsonia naganoensis* DSM 15166^{T}	73.4	72.6	20.0
GCF_014204935	*Conyzicola lurida* DSM 105784^{T}	73.4	73.0	20.4
GCF_900167575	*Okibacterium fritillariae* VKM Ac-2059^{T}	73.3	72.6	20.4
GCF_000470775	*Leifsonia xyli subsp. cynodontis* DSM 46306^{T}	73.2	72.5	20.1
GCF_020009625	*Leifsonia poae* BS71^{T}	73.2	72.3	20.3
GCF_004570845	*Leifsonia flava* SYP-B2174^{T}	73.2	72.6	20.5
GCF_014639655	*Conyzicola nivalis* CGMCC 1.12813^{T}	73.2	72.8	20.5
GCF_013408745	*Leifsonia soli* DSM 23871^{T}	72.9	72.4	19.8
GCF_006716125	*Homoserinimonas aerilata* DSM 26477^{T}	72.9	72.4	20.9
GCF_004801905	*Glaciibacter flavus* YIM 131861^{T}	72.8	72.4	20.8
GCF_000421145	*Glaciibacter superstes* DSM 21135^{T}	72.7	71.9	20.4
GCF_013410665	*Leifsonia psychrotolerans* LI1^{T}	72.5	71.9	20.3
GCF_014645015	*Salinibacterium xinjiangense* CGMCC 1.5381^{T}	71.8	71.2	20.5
GCF_003113315	*Salinibacterium hongtaonis* S1194^{T}	71.8	71.2	20.4
GCF_014652955	*Salinibacterium amurskyense* KCTC 9931^{T}	71.4	7.1	19.5
GCF_006788895	*Rhodoglobus vestalii* DSM 21947^{T}	70.4	69.5	21.4
GCF_000477555	*Leifsonia rubra* CMS 76R^{T}	70.4	69.6	20.6

Table S3. Presence of secondary metabolite biosynthetic gene clusters in the genome sequence of strain AK-R2A1-2^T, as detected using antiSMASH.

Region	From	To	smBGC type	Most similar known cluster	Similarity (%)	Core biosynthetic gene(s)	Additional biosynthetic gene(s)
Region 1	940,093	974,349	NAPAA	-	-	1	0
Region 2	1,478,451	1,502,396	Beta-lactone	Microansamycin (Polyketide)	7	2	1
Region 3	2,015,037	2,056,128	T3PKS	Alkylresorcinol (Polyketide)	66	1	0
Region 4	2,692,066	2,709,921	RRE-containing	Kosinostatin (NPR + Polyketide)	-	1	0
Region 5	3,035,049	3,068,677	Beta-lactone	-	-	3	7
Region 6	3,136,010	3,156,939	Terpene	Carotenoid (Terpene)	50	1	0
Region 7	3,218,696	3,243,543	Redox-cofactor	Lipopolysaccharide (Saccharide:Lipopolysaccharide)	5	3	0
